# Clinical Outcomes and Co-Occurring Mutations in Patients with *RUNX1*-Mutated Acute Myeloid Leukemia

**DOI:** 10.3390/ijms18081618

**Published:** 2017-07-26

**Authors:** Maliha Khan, Jorge Cortes, Tapan Kadia, Kiran Naqvi, Mark Brandt, Sherry Pierce, Keyur P. Patel, Gautam Borthakur, Farhad Ravandi, Marina Konopleva, Steven Kornblau, Hagop Kantarjian, Kapil Bhalla, Courtney D. DiNardo

**Affiliations:** 1 Department of Leukemia, The University of Texas MD Anderson Cancer Center, Houston, TX 77030, USA; doc.maliha@gmail.com (M.K.); jcortes@mdanderson.org (J.C.); tkadia@mdanderson.org (T.K.); knaqvi@mdanderson.org (K.N.); mbrandt@mdanderson.org (M.B.); spierce@mdanderson.org (S.P.); gborthak@mdanderson.org (G.B.); fravandi@mdanderson.org (F.R.); mkonople@mdanderson.org (M.K.); skornblau@mdanderson.org (S.K.); hkantarjian@mdanderson.org (H.K.); kbhalla@mdanderson.org (K.B.); 2 Department of Hematopathology, The University of Texas MD Anderson Cancer Center, Houston, TX 77030, USA; kppatel@mdanderson.org

**Keywords:** *RUNX1*, mutations, acute myeloid leukemia, hypomethylating agents, chemotherapy, prognosis

## Abstract

(1) Runt-related transcription factor 1 (*RUNX1*) mutations in acute myeloid leukemia (AML) are often associated with worse prognosis. We assessed co-occurring mutations, response to therapy, and clinical outcomes in patients with and without mutant *RUNX1* (*mRUNX1*); (2) We analyzed 328 AML patients, including 177 patients younger than 65 years who received intensive chemotherapy and 151 patients >65 years who received hypomethylating agents. *RUNX1* and co-existing mutations were identified using next-generation sequencing; (3) *RUNX1* mutations were identified in 5.1% of younger patients and 15.9% of older patients, and were significantly associated with increasing age (*p* = 0.01) as well as intermediate-risk cytogenetics including normal karyotype (*p* = 0.02) in the elderly cohort, and with lower lactate dehydrogenase (LDH; *p* = 0.02) and higher platelet count (*p* = 0.012) overall. Identified co-occurring mutations were primarily *ASXL1* mutations in older patients and *RAS* mutations in younger patients; FLT3-ITD and IDH1/2 co-mutations were also frequent. Younger *mRUNX1* AML patients treated with intensive chemotherapy experienced inferior treatment outcomes. In older patients with AML treated with hypomethylating agent (HMA) therapy, response and survival was independent of *RUNX1* status. Older *mRUNX1* patients with prior myelodysplastic syndrome or myeloproliferative neoplasms (MDS/MPN) had particularly dismal outcome. Future studies should focus on the prognostic implications of *RUNX1* mutations relative to other co-occurring mutations, and the potential role of hypomethylating agents for this molecularly-defined group.

## 1. Introduction

The genetic diversity of acute myeloid leukemia (AML) and the complexity of its multi-step pathogenesis now allow the classification of AML based on molecular events. The evolution of AML has traditionally been proposed to follow the “two-hit model” [1], in which two classes of mutations are required for cancer development. Class I mutations are activating mutations that stimulate cell survival and proliferation, while class II mutations are inactivating mutations that interfere with hematopoietic differentiation [2]. Runt-related transcription factor 1 (*RUNX1*) is a key hematopoietic transcription factor that regulates genes involved in myeloid differentiation, and is generally considered to be a classical tumor suppressor (class II) mutation [2]. Mutations of *RUNX1* are reported in approximately 10–16% of AML patients [3,4] and 12–24% of myelodysplastic syndrome (MDS) patients [5,6]. *RUNX1* alterations predominate in the morphologically undifferentiated French-American-British (FAB) M0 subtype. Clinical features associated with this mutation include older age, male sex, and absence of cytogenetic abnormalities [4].

In patients with AML, mutant *RUNX1* often associates with certain class I mutations. Gene expression profiling has identified the co-occurrence of *RUNX1* with mutations of the chromatin remodeling gene *ASXL1* or partial tandem duplication of the transcription regulator *MLL* in all major cohorts to date, while *RUNX1* and *NPM1* (nucleophosmin) mutations are consistently negatively correlated [2,7]. *ASXL1* mutations appear to be the most frequent co-mutation with *RUNX1*, and *ASXL1/RUNX1* double mutants are associated with lower rates of therapeutic response [8,9]. Other significant associations, such as with alterations in the tyrosine kinase *FLT3*, splicing factor mutations, or isocitrate dehydrogenase (*IDH1* or *IDH2*) has not been firmly established [3,4,7,10]; however, concomitant *FLT3* mutations are thought to play a synergistic role with *RUNX1* mutations in the development of AML [11]. Upregulation of genes normally expressed in hematopoietic progenitor cells or lymphoid cells, and downregulation of promoters of myelopoeisis also ascribe a unique gene expression signature to *RUNX1*-mutated AML [3,12], implicating upregulation of oncogenic pathways such as BCR, TLR-4 and NOTCH1 to the pathogenicity of mutant *RUNX1*.

Although standard intensive chemotherapy has yielded high remission rates and superior long-term survival in younger AML patients [13], elderly patients continue to experience lower response rates and poor long-term outcomes [14], and hypomethylating agents (HMAs) are typically utilized in the older patient population [14]. Clinical outcomes with respect to the influence of *RUNX1* mutations based on age and treatment regimen have not been previously explored [4].

*RUNX1* mutations are correlated with poor clinical outcomes. Gaidzik et al. [10] compared 53 mutant *RUNX1* and 831 wild-type *RUNX1* newly-diagnosed AML patients, and found inferior rates of event-free survival (EFS), relapse-free survival (RFS), and overall survival (OS) in *RUNX1*-mutated patients in patients 60 years of age or younger and treated with intensive chemotherapy (EFS, 8% vs. 30%; RFS, 26% vs. 44%; OS, 32% vs. 45%). In an analysis of AML patients treated with intensive chemotherapy by Tang et al. [7], the complete remission rate was lower in 62 newly-diagnosed patients with *RUNX1*-mutated AML compared with 408 without the mutation (56.8% vs. 77.5%). A statistically higher incidence of induction-related death in patients with mutant-*RUNX1* AML (10.8% vs. 6.5%) was also identified. Other studies have also correlated *RUNX1* mutations with resistance to chemotherapy and higher rates of refractory disease [2,7,10]. It is important to expand our understanding of the clinical outcomes of mutant and wild-type *RUNX1* in relation to specific treatment modalities, with attention to the older patient population treated with hypomethylating or lower-intensity approaches where *RUNX1* mutations are more often identified.

This study examined the frequency of *RUNX1* mutations in newly diagnosed patients with AML, and their effect on clinical outcomes and treatment response rates, including younger patients receiving chemotherapy and elderly patients receiving HMAs. Co-existing mutations and their effect on the clinical course of AML in conjunction with *RUNX1* were also examined. 

## 2. Results

### 2.1. Frequency and Characteristics of RUNX1 Mutations

Overall, mutant *RUNX1* was identified in 33 (10.1%) patients with newly diagnosed AML. These rates were significantly higher in patients 65 years of age or older, 24 (15.9%), compared with the younger adult cohort, 9 (5.1%). None of the patients were identified as having more than one pathogenic *RUNX1* mutation at diagnosis. The majority (>90%) of *RUNX1* mutations described were either frameshift or nonsense in nature, resulting in early amino acid chain termination and truncated proteins. The specific characteristics of these mutations, including their allelic frequencies, types, and exon locations, have been elaborated in Appendix A Table A1. 

### 2.2. Association of RUNX1 with Clinical Characteristics

Table 1 summarizes the clinicopathologic variables evaluated with respect to the impact of *RUNX1* mutational status. In the elderly cohort, a significant correlation was observed between the presence of mutated *RUNX1* and increasing age (*p* = 0.01); *mRUNX1* was rare in the younger patient cohort (5%). Along with an increasing age, *RUNX1* mutations occurred more frequently in older patients with intermediate-risk cytogenetics (particularly those with a normal karyotype), as compared to those with complex cytogenetic abnormalities (*p* = 0.02). Lower lactate dehydrogenase (LDH) levels and higher platelet counts were also found to be significant in *RUNX1*-mutated patients within the entire cohort overall (*p* = 0.02 and *p* = 0.012 respectively). *RUNX1* mutational status did not significantly correlate with WBC count, hemoglobin, or peripheral blood and bone marrow blasts, or history of prior myelodysplastic syndrome or myeloproliferative neoplasms (MDS/MPN) or therapy-related AML (t-AML). Among the mutant *RUNX1* patients, 15.2% (*n* = 20) of the patients classified as FAB M0 subtype.

### 2.3. Association of RUNX1 with Co-Occurring Mutations

In the older cohort, mutational analysis identified *ASXL1* mutations as the most frequent aberration in the presence of mutant *RUNX1*, co-occurring at a frequency of 37.5%—significantly higher compared to ASXL1 occurrence in wild-type *RUNX1* (14.2%) (*p* = 0.007). In comparison, the incidence of co-occurring *RAS* mutation was notable within the younger *mRUNX1* cohort, occurring in 4 of 9 patients (44.4%) (*p* = 0.184). The other most frequent co-occurring mutations in the younger cohort were those in *ASXL1*, *FLT3-ITD*, and *DNMT3A* genes at 33% each; compared to wild-type *RUNX1* (*ASXL1*, 2.4% *p* < 0.005; *FLT3-ITD*, 20.8% *p* = 0.379; *DNMT3A*, 20.2% *p* = 0.384). *IDH1* and *IDH2* mutations were also frequently identified in association with mutant *RUNX1*, with a frequency of mutant *IDH1* as 11.1% in *mRUNX1* compared to 4.8% wild-type (*p* = 0.407) in the younger cohort and 16.7% in *mRUNX1* compared to 12.6% in wild-type *RUNX1* in the elderly cohort (*p* = 0.603). *IDH2* mutations occurred at 22.2% (*mRUNX1*) and 15.5% (wild-type) (*p* = 0.617) and 20.8% (*mRUNX1*) compared to 16.5% (wild-type) (*p* = 0.624) in the younger and the elderly cohorts, respectively.

**Figure 1:**
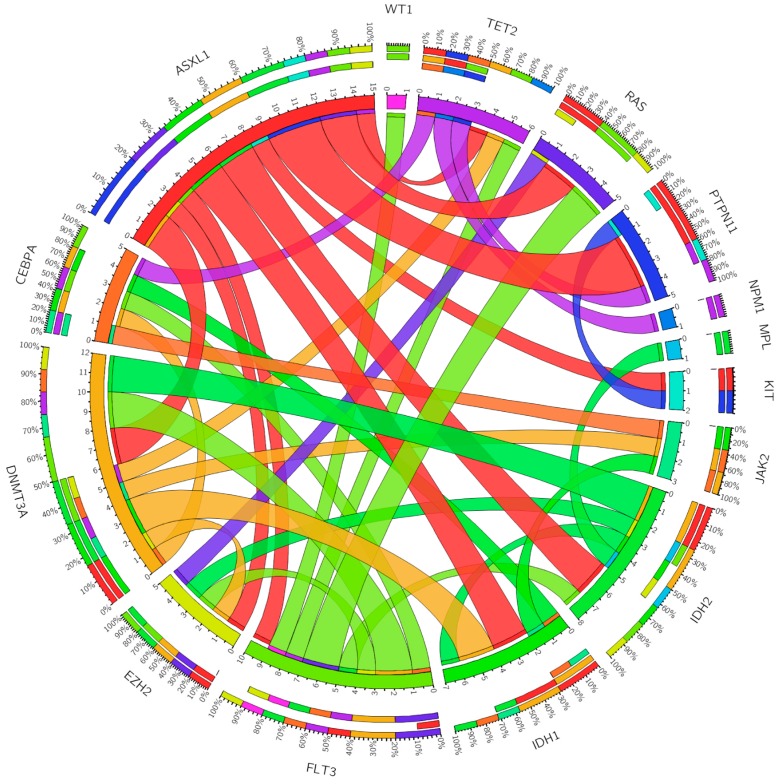
Co-occurring mutations among patients with *RUNX1* acute myeloid leukemia. The thickness of the connecting lines indicates the frequency with which the two mutations co-occur.

A number of other gene mutations were analyzed and showed varying association with mutated *RUNX1* (Figure 1) [15]. The frequencies of these co-mutations with mutant *RUNX1* in the younger and the elderly cohorts were, respectively, as follows: *FLT3-ITD*, 33.3% and 16.7%; *NPM1*, 0% and 4.2%; *RAS*, 44.4% and 8.3%; *IDH1*, 11.1% and 16.7%; *IDH2*, 22.2% and 20.8%; *TET2*, 0% and 12.5%; *PTPN11*, 11.1% and 8.3%; *JAK2*, 0% and 8.3%; *CEBPA*, 0% and 12.5%; *DNMT3A*, 33.3% and 16.7%; *EZH2*, 22.2% and 0%; *KIT*, 11.1% and 0%; *MPL*, 0% and 4.2%; *WT1*, 0% and 4.2%. Additionally, *MLL* translocation 11q23 was detected in four patients (1.2%) out of the total cohort of 328.

We additionally identified a number of mutations that were negatively associated with mutant *RUNX1*. Mutations in the *NPM1* gene were significantly lower in both the older (4.2%) and younger cohorts (0%) with *RUNX1* mutations, compared to those with wild-type *RUNX1* (older, 15.0%; younger, 26.2%). A similar negative correlation was identified between *RUNX1* and *CEBPα* mutations—particularly among the younger cohort, wherein CEBP*α* incidence with wild-type *RUNX1* was 16.1%, and was not identified (0%) in the setting of mutated *RUNX1*.

**Figure 2:**
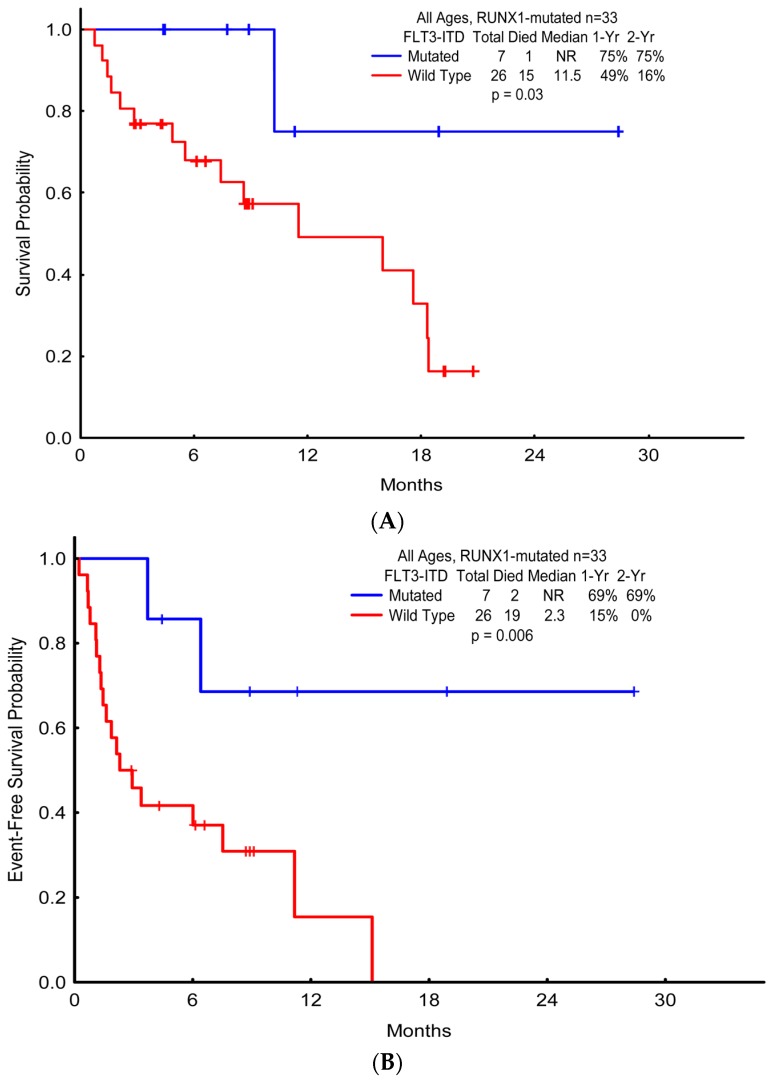
Survival probabilities of patients with *RUNX1* and *FLT3-ITD* mutations. (**A**) Overall survival; (**B**) Event-free survival.

**Figure A1:**
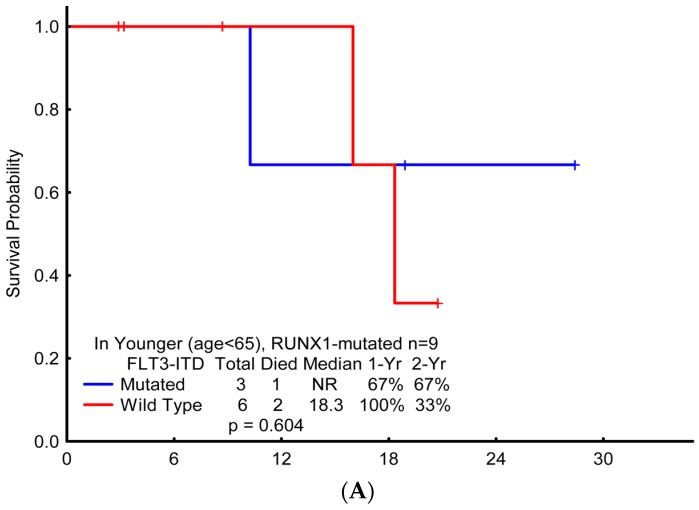

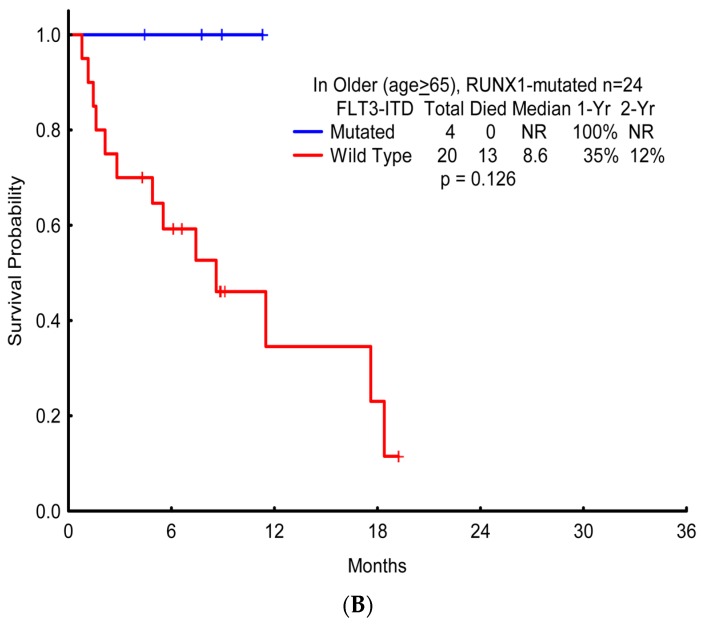
Survival probabilities of *FLT3-ITD* and *RUNX1* dual-mutated patients. (**A**) Patients aged below 65 years. (**B**) Patients older than 65 years.

*FLT3-ITD* mutations were frequently identified in both cohorts, irrespective of *mRUNX1*: frequencies in the presence of mutated *RUNX1* versus wild-type *RUNX1* (older, 16.7% vs. 15.7%; younger, 30.0% vs. 20.8%). Of interest, co-occurring *FLT3-ITD* in the setting of *mRUNX1* appeared to confer a more favorable prognosis (median OS with mutated *FLT3-ITD* vs. wild-type *FLT3-ITD*, NR vs. 11.5 months; *p* = 0.034) with a notable improved OS in *FLT3-ITD* and *RUNX1* dual-mutated patients (Figure 2), although these differences were not significant when separated by age (younger, *p* = 0.604; older, *p* = 0.126) (Figure A1). This overall prognostic effect was additionally observed with improved EFS in dual-mutated patients (median EFS with mutated *FLT-ITD3* vs. wild-type *FLT3-ITD*, NR vs. 2.3 months; *p* = 0.006).

### 2.4. Association of RUNX1 with Response to Frontline AML Therapy

In the elderly AML cohort, response to frontline therapy was generally unaffected by *RUNX1* mutational status (Table 2). There were no significant differences between patients with mutated and wild-type *RUNX1* for complete remission (CR) rates, overall response rate (ORR);(CR + complete remission with incomplete platelet recovery (CRp) + hematological improvement (HI) + partial remission (PR), HI, and/or early death (ED; death within 28 days of initiating treatment) in the cohort as a whole. Notably, a significant difference was observed for the subset of older AML patients with prior MDS/MPN (Table 3). Overall, ORR was obtained in 80% of MDS/MPN patients in the older cohort with wild-type *RUNX1*, but was achieved in only 25% of the patients identified with mutant *RUNX1* (*p* = 0.004).

In the younger cohort (Table 3), while CR rates were significantly reduced in patients with mutated versus wild-type *RUNX1* (33.3% vs. 76.2%; *p* = 0.004), no significant difference in overall response rate was observed.

### 2.5. Association of RUNX1 with Clinical Outcomes

**Figure 3:**
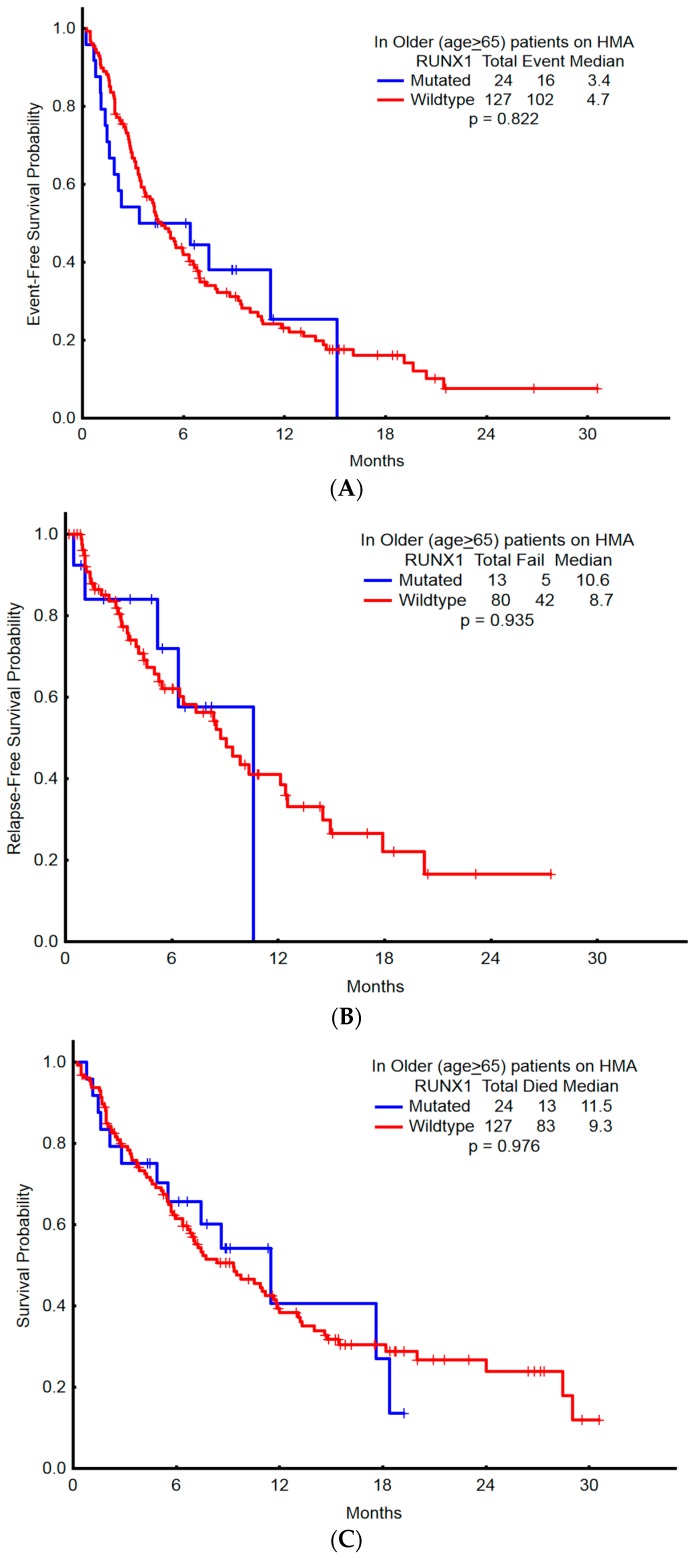
Survival probabilities among patients older than 65 years who received induction with hypomethylating agents, compared with the presence or absence of *RUNX1* mutation. (**A**) Overall survival; (**B**) Relapse-free survival; (**C**) Event-free survival.

**Figure 4:**
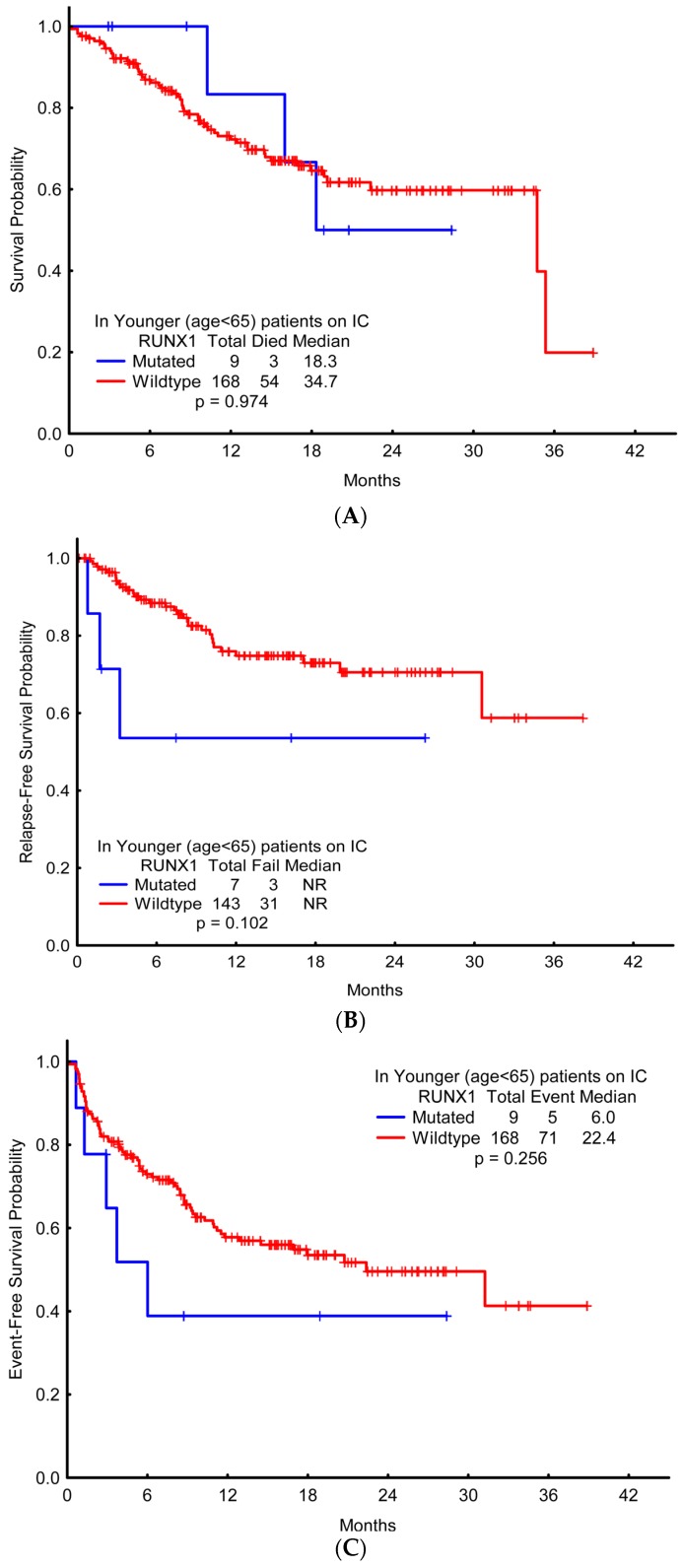
Survival probabilities among patients aged below 65 years who received induction with chemotherapy, compared with the presence or absence of *RUNX1* mutation. (**A**) Overall survival; (**B**) Relapse-free survival; (**C**) Event-free survival.

**Figure 5:**
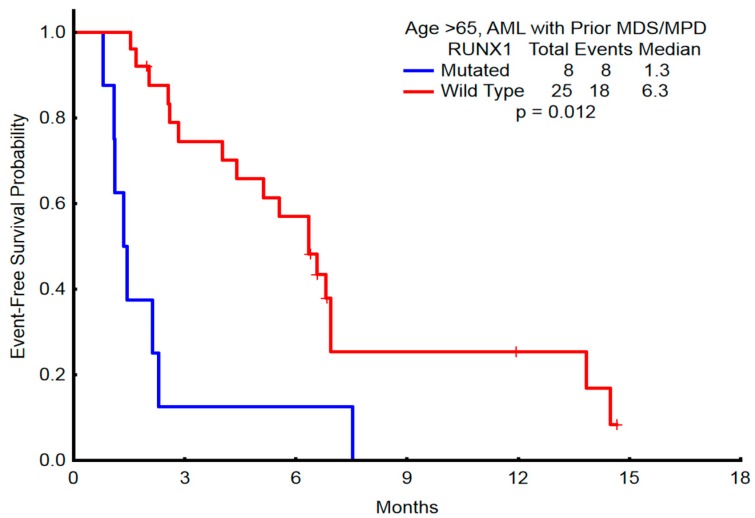
Event-free survival in patients with prior MDS/MPN who were older than 65 years and received HMA induction therapy.

OS, EFS, and RFS were not significantly different between mutant *RUNX1* compared with wild-type (Figure 3 and Figure 4). In the younger cohort, the two-year OS with wild-type *RUNX1* and mutant *RUNX1*, respectively, was 60% and 50% (95% CI: ± 13% vs. ±98%); two-year RFS was 71% and 54% (95% CI: ±15% vs. ±85%); two-year EFS was 50% and 39% (95% CI: ±13% vs. ±85%). The clinical outcomes of the older cohort showed similar results, with no significant difference observed between the OS, EFS, and RFS of mutated-*RUNX1* patients versus that of wild-type-*RUNX1* patients. In a subgroup analysis of patients with a history of prior MDS/MPN (*n* = 40), those with mutant *RUNX1* had significantly shorter EFS compared with wild-type *RUNX1* (1.35 vs. 6.34 months; *p* = 0.012) (Figure 5) and trend to inferior OS (*p* = 0.079) in the older patient cohort. 

## 3. Discussion

In our study, *RUNX1* mutations were detected at an overall incidence of 10.1%. This is consistent with previous studies that reported mutated *RUNX1* alleles in 5.6% to 16.1% of patients with AML [3,4,7,10,12].

Mutations in *RUNX1* associate with increasing age, also consistent with previous studies examining *RUNX1* mutation status and age [4,7,12,16]. Accumulation of cellular stress and impaired repair of double-stranded DNA breaks (e.g., after radiation exposure) may contribute to genomic instability [17] and increase the frequency of genetic mutations, including *RUNX1* [18,19,20]. Furthermore, *RUNX1* mutations in our elderly cohort clustered among patients identified with intermediate cytogenetic risk including cytogenetically-normal AML, consistent with previous studies [7,10]. Other clinical characteristics (white blood cell (WBC) count, hemoglobin, peripheral blood and bone marrow blasts, t-AML, and AML secondary to MDS/MPN) were not associated with *RUNX1* mutational status. Our analysis additionally confirmed a relationship between *RUNX1* mutation and platelet count and LDH level, as has been previously reported [4,7].

The most commonly co-occurring mutation overall and particularly prevalent in the older cohort was ASXL1. Of interest, clonal expansion of *ASXL1* is one of the most frequently identified genes in age-related clonal hematopoiesis, and subsequent *RUNX1* mutations may be implicated in malignant progression in these individuals [21,22]. Among younger patients, co-occurring *RAS* mutations (44%) and FLT3-ITD (33%) were also frequent. Their classification as class I mutations represents their capacity to promote stem cell proliferation and thereby cooperate with *RUNX1* mutations in AML pathogenesis [2,23]. 

Co-occurring *FLT3* and *RUNX1* mutations have been previously described, with a hypothesis that loss of function *RUNX1* mutations may predispose hematopoietic cells to overexpress activating mutations of *FLT3* [24]. When examining the clinical implications of this hypothesis in patients with mutant *RUNX1*, our analysis interestingly found superior OS and EFS in *mRUNX1* associated with *FLT3* mutations. Previous studies have identified that the adverse effect of *RUNX1* is independent of FLT3-ITD [25]. Evaluation in future studies will be important to see if this is a reproducible finding. It is also important to note that *NPM1* and biallelic *CEBPα* mutations confer a favorable prognosis in terms of disease-free survival and OS in the absence of *FLT3* mutations [2], and both are inversely related to the incidence of *RUNX1* mutations [7]. In our cohort, the incidence of *NPM1* mutations declined from 26.2% to 0% in younger patients and from 15.0% to 4.2% in older patients in the absence and presence of *RUNX1* mutations, respectively. Conversely, although partial tandem duplications in the *MLL* gene have been previously associated with mutated *RUNX1* and implicated in shorter survival [2,7], this association was not identified in our patient cohort, as only four patients were identified with the *MLL* rearrangement 11q23. An association of co-occurring *IDH1*/*IDH2* mutations with *RUNX1* mutations was additionally identified, consistent with Gaidzik et al. [10]. Furthermore, mutations in *RUNX1* and *IDH1/2* have been collectively described as the most frequent genetic lesions in M0-AML [26], the prognostic impact of the latter remaining controversial [27].

While *RUNX1* mutations are frequently associated with inferior responses to AML therapy, our analysis suggests that the poor outcomes seen in *RUNX1* mutations occur primarily within younger patients treated with intensive therapeutic strategies, as well as the subgroup of *RUNX1* mutant AML with prior MDS/MPN. The similar responses and outcome of the larger cohort of older *mRUNX1* treated with HMA approaches was a notable finding of this analysis.

A previous study in patients with MDS identified that treatment with HMAs may partially mitigate the adverse prognostic impact of gene mutations in patients with *RUNX1*, *ASXL1*, *EZH2*, or *ETV6* mutations [28]. Differences in outcomes between patient populations may also be linked with the distribution of co-existing mutations and cytogenetic abnormalities that may exacerbate or mitigate the effect of mutated *RUNX1*, [2] and our study is limited by a lack of complete co-mutational data, most notably for splicing factor mutations, as whole exome sequencing was not possible. The importance of *RUNX1* as a marker of MRD in responding patients was additionally not able to be assessed in our study. 

## 4. Materials and Methods

### 4.1. Patients

A total of 328 patients with treatment-naïve AML treated at the University of Texas MD Anderson Cancer Center between August 2013 and July 2016 were analyzed in this study. The presence of de novo AML, or history of prior myelodysplastic syndrome (MDS) or myeloproliferative neoplasms (MPNs) was collected. As treatment intensity at our institution during this time period was strongly and near-universally correlated to patient age at diagnosis, patients were categorized into two cohorts according to their age and therapeutic regimen: patients younger than 65 years who received intensive chemotherapy (younger cohort; *n* = 177) and patients ≥65 years who received HMA therapy (elderly cohort; *n* = 151).

Variables in our study included patient demographics (age and sex), relevant diagnostic tests including white blood cell (WBC) count, hemoglobin (Hb) levels, platelet count, lactate dehydrogenase (LDH) levels, and percentage of bone marrow blasts, cytogenetics, FAB classification, *RUNX1* mutation, and co-occurrence of other mutations. Clinical outcomes were assessed using Kaplan–Meier estimates of OS, EFS, and RFS [29]. Response to therapy was evaluated using revised IWG criteria for AML [30].

All participating patients provided informed consent in compliance with the Declaration of Helsinki, and the study received ethical approval by the Institutional Review Board as part of a retrospective chart review protocol, PA11.0788 on 10 October 2011 within the Department of Leukemia at University of Texas MD Anderson Cancer Center.

### 4.2. Molecular Analysis

Diagnostic bone marrow samples were obtained for mutational analysis. Total genomic DNA was extracted from unenriched peripheral blood (PB) or bone marrow (BM) samples using ReliaPrep genomic DNA isolation kit (Promegal Corp, Madison, WI, USA) [31]. *FLT3* (internal tandem duplication and D835) was assessed by PCR followed by capillary electrophoresis on a Genetic Analyzer (Applied Biosystems, Foster City, CA, USA) [32]. Briefly, a total of 250 ng DNA was utilized to prepare sequencing libraries using Agilent HaloPlex custom Kit (Agilent Technologies, Santa Clara, CA, USA) [33]. The entire coding sequences of 28 genes (*ABL1*, *ASXL1*, *BRAF*, *DNMT3A*, *EZH2*, *FLT3*, *GATA1*, *GATA2*, *HRAS*, *IDH1*, *IDH2*, *IKZF2*, *JAK2*, *KIT*, *KRAS*, *MDM2*, *MLL*, *MPL*, *NPM1*, *NRAS*, *PTPN11*, *RUNX1*, *TET2*, *TP53*, *WT1*) were interrogated on a custom-designed next-generation sequencing approach using the IlluminaMiSeq platform (Illumina; San Diego, CA, USA) [34]. The genomic reference sequence used was genome GRch37/hg19. The following software tools were utilized in the experimental setup and data analysis: Illumina Experiment Manager 1.6.0 (Illumina; San Diego, CA, USA), MiSeq Control Software 2.4 (Illumina; San Diego, CA, USA), Real Time Analysis 1.18.54 (Illumina; San Diego, CA, USA), Sequence Analysis Viewer 1.8.37 (Illumina; San Diego, CA, USA), MiSeq Reporter 2.5.1 (Illumina; San Diego, CA, USA), and SureCall 3.0.1.4 (Agilent Technologies; Santa Clara, CA, USA). A minimum of 80% reads at quality scores of AQ30 or higher were required to pass quality control. NM_001754 was utilized as the *RUNX1* reference sequence. 

The lower limit of detection of this assay (analytical sensitivity) for single nucleotide variations was determined to be 5% (one mutant allele in the background of nineteen wild type alleles) to 10% (one mutant allele in the background of nine wild type alleles). Testing of patients with active hematologic malignancies was limited to somatic mutations only. Known germline polymorphisms identified in >20% of our in-house patient population were excluded. Additionally, germline polymorphisms previously reported in population databases such as dbSNP and ExAC were classified as “variants of probable germline origin” and were excluded from this analysis. 

### 4.3. Statistical Analysis

Continuous variables were described as means, medians, standard deviations, and ranges, whereas the categorical variables were presented as frequencies and percentages. The categorical variables in patients with mutated *RUNX1* were compared with the wild-type using the Chi-squared test. Kaplan–Meier curves [26] were used to estimate unadjusted OS durations. OS was calculated from the start of treatment to the last follow up or death (censor = alive/dead). EFS was measured from the start of treatment to any adverse event or the last follow-up. Adverse events included either relapse (i.e., NR, ED) or death. RFS was determined from leukemia-free response (i.e., at the time of documented CR, CRp, CRi, PR) to relapse (loss), death, or last follow-up. For all analyses, *p* < 0.05 was considered statistically significant. All computations were carried out in Statistica 12.0 (StatSoftInc. Tulsa, OK, USA; 1984–2013).

## 5. Conclusions

In conclusion, *RUNX1* mutations are among the most frequent recurring genetic abnormalities in patients with AML, often associated with specific clinical features and poor outcomes. *ASXL1*, *DNMT3A*, *IDH1/2*, *FLT3-ITD*, and *RAS* mutations frequently co-occur with *RUNX1* mutations. *FLT3-ITD* mutations did not impart an inferior prognosis in *mRUNX1* AML, and of note appeared to confer an improved OS in this molecularly defined cohort. While younger *mRUNX1* patients treated with intensive chemotherapy and a subgroup of older *mRUNX1* patients with prior MDS/MPN exhibited inferior treatment responses, the majority of *mRUNX1* AML patients included older patients treated with HMA therapy, in whom treatment responses and clinical outcomes were not inferior compared to *RUNX1* wild-type. Further studies investigating the treatment response to HMAs—in particular in younger patient populations with *RUNX1*-mutated AML—are required to fully establish their therapeutic role in the individualized treatment of these patients.

## Figures and Tables

**Table 1:** Clinical characteristics in the younger cohort receiving chemotherapy and the elderly cohort receiving hypomethylating agents according to *RUNX1* mutation status.

Clinical Characteristics	Younger Cohort (Chemotherapy)		*p*-Value	Elderly Cohort (HMA)		*p*-Value
	Median (Range)			Median (Range)		
	*RUNX1^mut^*	*RUNX1^WT^*		*RUNX1^mut^*	*RUNX1^WT^*	
	(*n* = 9)	(*n* = 168)		(*n* = 24)	(*n* = 127)	
Age, year	56 (31–63)	51 (17–64)	0.14	77 (65–92)	73 (65–91)	0.01 *
WBC, ×10^9^/L	4.95 (1.3–17.2)	6.9 (0.5–378.4)	0.43	3.2 (0.6–36.5)	3.2 (0.3–164.5)	0.98
Platelet count, ×10^9^/L	57.5 (28–213)	30 (1–584)	0.05	61 (7–416)	42 (3–324)	0.20
Hemoglobin, g/dL	8.95 (7.4–12.1)	9.3 (5.1–13.0)	0.38	9.4 (5.8–13.1)	9.4 (7.5–13.2)	0.37
Peripheral blood blasts, %	28 (1–86)	25.5 (0–97)	0.61	7 (0–90)	11 (0–95)	0.51
Neutrophils, %	14.5 (0–45)	12 (0–98)	0.93	21 (0–81)	21 (0–73.3)	0.95
Bone marrow blasts, %	51 (28–93)	54 (2–96)	0.66	50 (12–84)	44 (1–90)	0.80
Cytogenetic			0.94			0.02
Complex	2	37		2	47	
Diploid	3	65		14	50	
^ Intermediate	4	66		8	30	
LDH, U/L	668.5	888	0.10	532	650	0.44
	(526–1649)	(310–12,489)		(276–2314)	(210–3921)	
t-AML	0	10	0.45	4	18	0.75
Prior MDS/MPD	1	6	0.26	8	25	0.14

Abbreviations: LDH, lactate dehydrogenase; HMA, hypomethylating agent; Prior MDS/MPD: Prior myelodysplastic syndrome or myeloproliferative neoplasms; t-AML, therapy-related acute myeloid leukemia; WBC, white blood cell; * Boldface indicates statistical significance. ^ Intermediate also includes insufficient metaphases and not done.

**Table 2:** Clinical outcomes of all patients according to age, treatment, and *RUNX1* mutation status.

Response	Younger Cohort (Chemotherapy)			Elderly Cohort (HMA)		
	*n* (%)			*n* (%)		
	*RUNX1^WT^*	*RUNX1^mut^*	*p*-Value	*RUNX1^WT^*	*RUNX1^mut^*	*p*-Value
	(*n* = 168)	(*n* = 9)		(*n* = 127)	(*n* = 24)	
Complete remission	128 (76.2)	3 (33.3)	0.004 *	48 (37.8)	9 (37.5)	0.976
Complete remission with incomplete platelet recovery	9 (5.4)	3 (33.3)	0.271	11 (8.7)	1 (4.2)	0.667
Hematological improvement	4 (2.4)	1 (11.1)	0.631	17 (13.4)	3 (12.5)	0.603
Partial remission	2 (1.2)	0 (0.0)		4 (3.1)	0 (0.0)	
Overall response rate	143 (85.0)	7 (78.0)	0.549	80 (63.0)	13 (54.0)	0.418
No remission	19 (11.3)	2 (22.2)		22 (17.3)	7 (29.2)	
Death	5 (3.0)	0 (0.0)		22 (17.3)	3 (12.5)	
Early death **	1 (0.6)	0 (0.0)	0.818	3 (2.4)	1 (4.2)	0.617

* Boldface indicates statistical significance. Early death **: Death within 28 days of initiating treatment.

**Table 3:** Clinical outcomes of patients with prior MDS or MPD according to age, treatment, and *RUNX1* mutation status.

Response	Younger Cohort (Chemotherapy)			Elderly Cohort (HMA)		
	*n* (%)			*n* (%)		
	*RUNX1^WT^*	*RUNX1^mut^*	*p*-Value	*RUNX1^WT^*	*RUNX1^mut^*	*p*-Value
	(*n* = 6)	(*n* = 1)		(*n* = 25)	(*n* = 8)	
Complete remission	2 (33.3)	1 (100.0)	0.211	11 (44.0)	1 (12.5)	0.107
Complete remission with incomplete platelet recovery	1 (16.7)	0 (0.0)		4 (16.0)	0 (0.0)	
Hematological improvement	0 (0.0)	0 (0.0)		4 (16.0)	1 (12.5)	
Partial remission	1 (16.7)	0 (0.0)		1 (4.0)	0 (0.0)	
Overall response rate	4 (66.7)	1 (100.0)	0.497	20 (80.0)	2 (25.0)	0.004 *
No remission	1 (16.7)	0 (0.0)		1 (4.0)	3 (37.5)	
Death	1 (16.7)	0 (0.0)		4 (16.0)	2 (25.0)	
Early death **	0 (0.0)	0 (0.0)		0 (0.0)	1 (12.5)	

* Boldface indicates statistical significance. Early death **: Death within 28 days of initiating treatment.

## Appendix A

**Table A1:** *RUNX1* mutations according to their minor allele frequency, exon location, and mutation type.

Mutation HGVS (MAF%)	Exon	Type
NM_001754.4 ( *RUNX1*):c.497G>A p.R166Q [46.4]	5	Missense
NM_001754.4 ( *RUNX1*):c.423_424insAAAGGGATTCT p.A142fs [5.9]	5	Frameshift
NM_001754.4 ( *RUNX1*):c.891_894dupCCCA p.A299fs [8.7]	8	Frameshift
NM_001754.4 ( *RUNX1*):c.238dupG p.E80fs [50.3]	4	Frameshift
NM_001754.4 ( *RUNX1*):c.900_901insTCCG p.P301fs [17.0], NM_001754.4 (*RUNX1*):c.987_988dupGT p.F330fs [32.3], NM_001754.4(*RUNX1*):c.1281_1285del p.I428fs [13.6]	“8,9”	Frameshift
NM_001754.4 ( *RUNX1*):c.292delG p.L98fs,NM_001754.4(*RUNX1*):c.492_493insAT p.G165fs [36.2]	“4,5”	Frameshift
NM_001754.4 ( *RUNX1*):c.368A>C p.D123A [9.2], NM_001754.4(*RUNX1*):c.367G>C p.D123H [9.3], NM_001754.4 (*RUNX1*):c.361_364del p.L121fs [10.1]	5	Frameshift
NM_001754.4( *RUNX1*):c.422_423insA p.A142fs [26.2]	5	Frameshift
NM_001754.4 ( *RUNX1*):c.418_422dupTACTC p.A142fs [21.0]	5	Frameshift
NM_001754.4 ( *RUNX1*):c.422dupC p.A142fs [9.5], NM_001754.4(*RUNX1*):c.424_425insGGTGG p.A142fs [4.4]	5	Frameshift
NM_001754.4 ( *RUNX1*):c.939_942dupCTCT p.A315fs [43.5]	8	Frameshift
NM_001754.4 ( *RUNX1*):c.593A>G p.D198G [83.0]	6	Missense
NM_001754.4 ( *RUNX1*):c.592G>A p.D198N [31.7]	6	Missense
NM_001754.4 ( *RUNX1*):c.993_1005del p.D332fs	9	Frameshift
NM_001754.4 ( *RUNX1*):c.402dupT p.G135fs [41.1]	5	Frameshift
NM_001754.4 ( *RUNX1*):c.1023_1024insC p.I342fs	9	Frameshift
NM_001754.4 ( *RUNX1*):c.1251_1252insC p.M418fs	9	Frameshift
NM_001754.4 ( *RUNX1*):c.1244_1250dupAGTTCTC p.M418fs [19.5]	9	Frameshift
NM_001754.4 ( *RUNX1*):c.548dupC p.P184fs [45.3]	6	Frameshift
NM_001754.4 ( *RUNX1*):c.256_259delinsACCCGA p.P86fs	4	Missense
NM_001754.4 ( *RUNX1*):c.484A>G p.R162G [70.4]	5	Missense
NM_001754.4 ( *RUNX1*):c.485G>A p.R162K [42.2],NM_001754.4(*RUNX1*):c.388G>T p.V130F [44.7]	5	Missense
NM_001754.4 ( *RUNX1*):c.496C>T p.R166* [39.1]	5	Nonsense
NM_001754.4 ( *RUNX1*):c.601C>T p.R201* [40.7]	6	Nonsense
NM_001754.4 ( *RUNX1*):c.955_958dupAGTC p.R320fs [49.7]	8	Frameshift
NM_001754.4 ( *RUNX1*):c.952del p.S318fs [29.7]	8	Frameshift
NM_001754.4 ( *RUNX1*):c.1352_1356dupACGTG p.V453fs [22.9], NM_001754.4 (*RUNX1*):c.611G>A p.R204Q [49.7]	“9,6”	Frameshift
NM_001754.4 ( *RUNX1*):c.952T>G p.S318A [14.9], NM_001754.4 (*RUNX1*):c.954dupC p.S319fs [14.4], NM_001754.4(*RUNX1*) :c.950T>G p.L317R [15.0]	8	Frameshift
NM_001754.4 ( *RUNX1*):c.977del p.D326fs [17.1], NM_001754.4 (*RUNX1*):c.352-1_352insTTTAG p.?	9	Frameshift, splice
NM_001754.4 ( *RUNX1*):c.492_507dupCGGTCGAAGTGGAAGA p.G170fs [30.7], NM_001754.4 (*RUNX1*):c.484dupA p.R162fs [3.0]	5	Frameshift
NM_001754.4 ( *RUNX1*):c.486dupG p.F163fs [51.2]	5	Frameshift
NM_001754.4 ( *RUNX1*):c.1036dupC p.R346fs [2.8]	9	Frameshift
NM_001754.4 ( *RUNX1*):c.167T>C p.L56S [51.5]	4	Missense
